# The benefits of covariate adjustment for adaptive multi-arm designs

**DOI:** 10.1177/09622802221114544

**Published:** 2022-07-25

**Authors:** Kim May Lee, David S. Robertson, Thomas Jaki, Richard Emsley

**Affiliations:** 1 Institute of Psychiatry, Psychology and Neuroscience, King’s College London, London, UK;; 2 47959 MRC Biostatistics Unit, University of Cambridge, Cambridge, UK;

**Keywords:** Adaptive design, covariate adjustment, multi-arm, treatment selection, UMVCUE

## Abstract

Covariate adjustment via a regression approach is known to increase the precision of statistical inference when fixed trial designs are employed in randomized controlled studies. When an adaptive multi-arm design is employed with the ability to select treatments, it is unclear how covariate adjustment affects various aspects of the study. Consider the design framework that relies on pre-specified treatment selection rule(s) and a combination test approach for hypothesis testing. It is our primary goal to evaluate the impact of covariate adjustment on adaptive multi-arm designs with treatment selection. Our secondary goal is to show how the Uniformly Minimum Variance Conditionally Unbiased Estimator can be extended to account for covariate adjustment analytically. We find that adjustment with different sets of covariates can lead to different treatment selection outcomes and hence probabilities of rejecting hypotheses. Nevertheless, we do not see any negative impact on the control of the familywise error rate when covariates are included in the analysis model. When adjusting for covariates that are moderately or highly correlated with the outcome, we see various benefits to the analysis of the design. Conversely, there is negligible impact when including covariates that are uncorrelated with the outcome. Overall, pre-specification of covariate adjustment is recommended for the analysis of adaptive multi-arm design with treatment selection. Having the statistical analysis plan in place prior to the interim and final analyses is crucial, especially when a non-collapsible measure of treatment effect is considered in the trial.

## 1. Introduction

For some clinical areas, such as mental health and infectious diseases, there are many candidate interventions available. A key goal of an evaluation programme is to identify interventions that are beneficial to patients from the set of candidates. One way to achieve this goal is to conduct experiments through the different phases of clinical trials. A multi-arm design with many-to-one comparisons, i.e., comparing each candidate intervention with a control treatment, is one option for phase II and III studies. It is more efficient than conducting separate two-arm parallel trials on the same set of candidate interventions, since only a single control arm is recruited in a multi-arm trial.

Design adaptations have been proposed to further improve the efficiency of a multi-arm design based on accruing data collected during a study. The idea is known as an adaptive multi-arm design, which has at least one interim analysis, and the design structure can be considered as having multiple stages that are defined by the timing of interim analyses.^1,2^ For example, the features of adding and dropping arms reduce the duration of the evaluation process,^3,4^ and adaptive randomization can optimise a utility function that is of interest to stakeholders.^5–7^ The focus of this work is on the adaptation that achieves the “screening” (or treatment selection) goal with randomization probabilities being fixed in advance by design. Hereafter we use ‘adaptive multi-arm design’ (AMAD) to denote a multi-arm design that contains the pre-planned opportunity for such an adaptation.

Broadly, there are different types of design and inferential frameworks for AMADs.^8,9^ One framework is an extension of two-arm group-sequential designs where the rejection boundaries for early stopping (via hypothesis tests) are computed at the study planning stage.^10–13^ Another framework requires the pre-specification of rule(s) for treatment selection at the planning stage, and utilises a flexible testing approach for the final inference, such as the combination test approach^14–17^ or the error spending approach.^18^ Depending on the study context, AMADs have been known as drop-the-loser designs, pick the winner designs, multi-arm screening trials and adaptive seamless phase II/III designs.^8,19–22^ In any case, data collected from each stage is used in the final inference about the selected intervention(s); the inferential approach under each framework ensures that the family-wise error rate (FWER) is controlled at the desired level. The FWER is defined as the probability of falsely rejecting at least one null hypothesis, which is a generalisation of the type I error rate that is often controlled in two-arm designs.

Most of the existing literature about AMADs focuses on the operating characteristics of the design when different testing procedures are used.^23,24^ Some have also focused on the estimation of treatment effects ^25–31^ and confidence intervals,^32^ respectively, in the absence of covariates. This is because the standard estimation procedures that have been used in fixed designs may fail to have good properties (e.g. in terms of bias or coverage) when an AMAD is implemented.

One key feature of AMADs that has not been explored in the literature is the role of covariates in the design and analysis of such trials. In studies using fixed designs, i.e., non-adaptive designs, it is well-known that covariate adjustment increases the precision of the inference.^33–37^ To the best of our knowledge, only the work by Jaki and Magirr^38^ has briefly touched on the topic of covariate adjustment in the context of AMADs, but the work is based on the framework of group sequential designs. There are some publications that consider the role of covariates when other types of adaptation are implemented, such as the blinded sample size re-estimation method,^39,40^ the standard group-sequential design,^41^ and adaptive randomization.^42^ In this paper, we aim to fill this gap in the literature and explore the impact of covariate adjustment in AMADs with treatment selection. We show how covariate adjustment affects treatment selection rules, hypothesis testing and power, as well as the estimation of the treatment effects. For the latter, we derive the Uniformly Minimum Variance Conditionally Unbiased Estimator to account for covariate adjustment analytically. In addition, when the interest is in the odds ratios for binary endpoint, or the hazard ratios for time-to-event endpoint, it is often overlooked by researchers that covariate adjustment plays an important role in the definition of the estimand of interest. We evaluate the impact of covariate adjustments by simulation when a collapsible measure of treatment effect is considered for a continuous endpoint and revisit the concept of non-collapsiblity, emphasising the importance of considering conditional or unconditional treatment effects in the context of AMADs.

This paper is organised as follows. In Section 2, we present the design and the analysis of AMADs. In Section 3, we illustrate the treatment effect estimates following a linear regression model. In Section 4, we present a simulation study to examine the role of covariate adjustment in treatment selection and in the final inference. A case illustration based on the INHANCE study^43^ is then provided to depict the application of an AMAD framework. In Section 5, we discuss the issue when the odds ratio and hazard ratio are considered as the primary descriptive statistics in AMADs. In Section 6, we emphasise the practical aspects of implementing AMADs and give some concluding remarks.

## 2. Design and inference

Consider a two stage setting, i.e., where there is one interim analysis for treatment selection in an AMAD. We describe the treatment selection rule, hypothesis testing approach and the procedure for identifying the required total sample size. We present the standard estimators for a treatment effect as well as confidence intervals, noting that both of these may not be compatible with the testing framework due to the complexity in combining the stage-wise data in the testing framework.

### 2.1. Treatment selection rule of AMAD

In general, an AMAD has K+1 study arms at stage one and a subset of the intervention arms at stage two. The subset of interventions that continue to stage two together with the control group is identified according to a pre-specified treatment selection rule. Several treatment selection rules have been considered in the literature,^23,44,29^ which relate to the goal of the study. For instance, a threshold rule selects interventions to continue to the second stage together with the control treatment when their test statistics is greater than a pre-defined threshold. The size of the subset of interventions can range from 2 to K+1 , where the former corresponds to selecting only one active intervention and the latter corresponds to selecting all the initial interventions. The number of selected interventions can either be pre-defined in the selection rule or can be driven by the data of the initial stage. We emphasise that the control group continues in both stages of an AMAD such that contemporaneous control data is used in the final inference.

### 2.2. Hypothesis testing and sample size

When there are more than one primary research comparisons, the control of FWER is often required by regulators.^45–47^ A statistical framework to control for the FWER is required for an AMAD, since it is designed to screen multiple interventions at stage one (in a pre-defined way) and to test many-to-one comparisons at the end of the study (when more than one intervention is selected). Here we describe the combination test approach briefly. It is a flexible approach in the sense that other adaptations are allowed at interim analysis. For example, when the threshold rule is considered the number of selected interventions given stage one data is not known in advance; resources can be reallocated accordingly without inflating the FWER when the combination test approach is used in the final inference. This is the approach considered in the asd R package,^44^ which can be used to conduct simulation studies for AMADs, but in the absence of covariates.

Let $\mu_{k}$ denote the treatment effect measure that compares an intervention k with the control treatment, where k=1,…,K . Examples of treatment effect measures are mean difference for a continuous endpoint, risk difference for binary endpoint and the hazard ratio for survival outcome. A non-normally distributed treatment effect measure can often be transformed such that the standard Z -test or t -test can be implemented accordingly. For example, applying the logarithmic function to hazard ratios leads to an approximately normal distribution for the resulting parameter.

For superiority studies, the global null hypothesis in a multi-arm setting is $\mu_{1}=\ldots =\mu_{K}=0$ ; and the alternative hypothesis is that there is at least one treatment effect which is greater than zero. If there was only a single stage or all interventions continue to stage two with certainty, the classical Dunnett test^48^ can be applied to control for the FWER under this global null hypothesis.

When the design of a study is modified at interim analyses, a combination test can be applied to ensure that the FWER is controlled at the required level by combining the stage-wise p-values via a pre-specified function.^14^ For example, a Fisher’s combination test considers the product of the stage-wise p-values, whereas the weighted inverse normal method considers the summation of the weighted stage-wise p-values. When there is only one research comparison in a study, the stage-wise p-values from the standard Z-test or t-test are computed using the corresponding stage-wise data. The combined value is then compared with the α− level of the combination test for the decision to reject or not reject the hypothesis. When multiple hypotheses are tested, e.g., in the context of many-to-one comparisons, the stage-wise p-values can be computed from the classical Dunnett test using the corresponding stage-wise data.

In AMADs, some of the interventions do not continue to stage two of the study. For the comparison involving these interventions, only stage one data is available for the corresponding hypothesis test. In this context, the control of the FWER in the strong sense might be desirable, i.e., ensure that the probability of falsely rejecting at least one null hypothesis is less than the α− level under any configuration of true and false (elementary) null hypotheses. The closure test principle can be applied to achieve this goal, which states that an individual null hypothesis, $H_{0k^{\prime }}$ , is only rejected when the elementary hypothesis and all the associated intersection hypotheses, $H_{S}=\cap_{s\in S}H_{0s}$ where S⊆{1,…,K} with index sets that include $k^{\prime }$ are also rejected at the α− level. In other words, additional null hypotheses, i.e., the intersection hypotheses, are tested when the FWER is required to be controlled in the strong sense.

More specifically, the combination test is used to test $H_{S}$ where the stage-wise p-values are obtained from the classical Dunnett testing procedure that is used to test the intersection hypotheses (see for example Section 2.1 of Friede and Stallard ^23^). For intersection hypotheses that involve the deselected interventions, the stage one p-values of all the associated comparisons are considered, but only the second stage p-values of the comparisons involving the selected (and associated) interventions are considered in the combination test, since there is no data of the deselected interventions in stage two.^32^ Note that other testing procedures can be applied in place of the Dunnett test for the intersection hypotheses, such as Simes test, $\overset{˘}{S}$ idak test, and likelihood ratio tests (for normally distributed test statistics).^16,32^

For the power of an AMAD, different definitions can be considered when more than one intervention can be selected to continue to stage two.^49^ For example, conjunctive power is the probability of detecting all effective interventions whereas disjunctive power is the probability of detecting at least one effective intervention. Having specified the power, the choice of the combination test function and the testing procedures, one can conduct a simulation study to identify the required sample size under the (global) alternative hypothesis in an iterative manner.

One approach is to vary the sample size per arm per stage, while another is to vary the overall sample size until the required power is obtained. Note that when the number of interventions to be selected to continue is not specified by the selection rule, the overall sample size of the former approach varies considerably across the trial replications. This may create uncertainty in costing if one does not plan to cover the maximum overall sample size. When the latter approach is considered, one needs to specify the total sample size per-stage in the simulation setting. In this case, when the stage-one total sample size is small (relative to the stage-two total sample size), the chance of selecting the truly effective interventions might be lower than the setting where the stage-one total sample size is larger. However, the latter setting can mean more subjects are unnecessarily exposed to the ineffective interventions when only a small subset of interventions are truly effective.

Having identified the required sample size for a given (global) alternative hypothesis, one can conduct a sensitivity analysis to examine the operating characteristics of an AMAD at the design stage. For example, one can vary the parameter configurations of treatment effects in the data generating mechanism (DGM) of the simulation to mimic other plausible scenarios. This will indicate how much power is gained or lost when the global alternative hypothesis does not hold.

### 2.3. Point estimation and confidence intervals

Estimating the treatment effects for AMADs is an additional important consideration, and one that has received relatively little attention in the literature compared with error rate control in hypothesis testing.^31^ A standard approach is to fit a regression model for the outcome/endpoint on the treatment group as well as the covariates of interest. The conventional point estimate of the treatment effect $\mu_{k}$ at the end of the trial is given by ${\hat{\beta }}_{k}$ , the estimated regression coefficient for treatment group k from the regression model fitted to all of the trial data. Note that there is less data from the deselected intervention arms than the selected arms. Similarly, at the interim analysis (i.e. the end of the first stage), the treatment effect estimate is given by ${\hat{\beta }}_{k}^{\left(1\right)}$ , the estimated regression coefficient for treatment group k from the model fitted to just the stage one data.

In general, given a selection rule based on the stage one data, the estimators ${\hat{\beta }}_{s}^{\left(1\right)}$ and ${\hat{\beta }}_{s}$ for a selected treatment s∈{1,…,K} will be conditionally *biased*, i.e. $E[{\hat{\beta }}_{s}^{\left(1\right)}]\neq \mu_{s}$ and $E[{\hat{\beta }}_{s}]\neq \mu_{s}$ . This is because a selected candidate intervention has to perform “well” according the the selection rule used in stage one in order to proceed to stage two, which leads to overly optimistic estimates of the treatment effect.^32,25^ When multiple interventions have similar effects, then the selected candidate is typically based on chance variability rather than true superiority.^50^ On the other hand, for the deselected interventions, the stage one regression estimate will be negatively biased due to early stopping.^32^ A simple unbiased estimator for a selected treatment effect $\mu_{s}$ is given by ${\hat{\beta }}_{s}^{\left(2\right)}$ , the estimated regression coefficient for treatment group s from the regression model fitted to just the stage two data. This estimator is unbiased since it is based on data post-selection. However, it is clearly inefficient since it ignores the stage one data. Hence unbiased and bias-adjusted estimators have been proposed in the adaptive designs literature,^25–29,51^ which aim to reduce or eliminate the conditional bias of the conventional end-of-trial estimate while still utilising all of the trial data. We give a concrete example of one such unbiased estimator in Section 3.2..

As for the construction of confidence intervals at the end of the trial, for many commonly-used regression models, the joint distribution of the estimated regression coefficients $\hat{\beta_{k}}$ , k=1,…,K , is (asymptotically) multivariate normal. This allows the construction of confidence intervals for $\mu_{k}$ in the usual way. In general, given a selection rule based on the stage one data, the confidence interval for a selected treatment effect $\mu_{s}$ constructed in this manner may not have the correct coverage probabilities. This is because the distribution of the estimator $\hat{\beta_{s}}$ is affected by the selection rule used and this is not taken into account. There have been some limited proposals for the construction of adjusted confidence intervals that take into account the treatment selection.^32,52^ However, in general these approaches can be very computationally intensive.

## 3. Normal endpoint with baseline covariate adjustment

We illustrate the estimators and their properties when baseline covariate adjustment is made in AMADs.

### 3.1. Regression models

Consider a normally distributed endpoint, $Y_{\;jk}^{\left(t\right)}$ , that is measured on patient j in arm k at stage t=1,2 , and two baseline covariates, $X1_{\;jk}^{\left(t\right)}$ and $X2_{\;jk}^{\left(t\right)}$ which may be correlated with the endpoint. Here j=1,2,…,J, denotes the index of patients in arm k , where k=0 for the control group and k=1,…,K for the intervention groups. We drop the superscripts (t) when referring to the data from the whole trial, i.e. pooling the observed outcome data from both stages. Note that if an arm k is dropped in stage one, we assume that there will not be any stage two data for the deselected arm. This is a potential concern when fitting regression models to the trial data, since if there is a time trend between stages this may not be accounted for.

To analyse the data at the end of the trial, one can fit the following linear regression model

(1) $$Y_{\;jk}=\alpha_{0}+\sum_{k=1}^{K}\beta_{k}\;I(T_{\;j}=k)+\gamma_{1}X1_{\;jk}+\gamma_{2}X2_{\;jk}+\epsilon_{\;jk}$$

where

-
$T_{\;j}$ is the study arm that patient j is randomized to;
-
$\beta_{k}$ are the stage-wise treatment contrasts between intervention k and the control treatment ( k=0 );
-
$\gamma_{1}$ is the effect of covariate X1 ;
-
$\gamma_{2}$ is the effect of covariate X2 ;
-
$\alpha_{0}$ is the model intercept;
-
$\epsilon_{\;jk}$ are iid $N(0,\sigma^{2})$ errors

Note that setting $\gamma_{1}=0$ corresponds to not adjusting for X1 , and similarly setting $\gamma_{2}=0$ corresponds to not adjusting for X2 . At the interim analysis one can fit the same model to only stage one data $Y_{\;jk}^{\left(1\right)}$ for the interim analysis where a pre-defined selection rule is used to select treatments for further study in stage two. For what follows, we also consider the same model fitted to only the stage two data $Y_{\;jk}^{\left(2\right)}$ .

### 3.2. Treatment effect estimators

The conventional point estimator at the end of the trial for the mean treatment effect of intervention k is given by ${\hat{\beta }}_{k}$ , which is the ordinary least squares (OLS) estimator arising from fitting model (1) to all of the trial data. As discussed in Section 2.3., given a selection rule based on the stage one data, this estimator will be conditionally biased. An unbiased but inefficient estimator is given by ${\hat{\beta }}_{k}^{\left(2\right)}$ , which is the OLS estimator from the model fitted to only the stage two data. For treatment selection at the interim analysis, let ${\hat{\beta }}_{k}^{\left(1\right)}$ denote the OLS estimator from the model fitted to the stage one data.

To derive an alternative unbiased estimator that utilises all of the trial data, the distributions of the stage-wise estimators ${\hat{\beta }}_{k}^{\left(1\right)}$ and ${\hat{\beta }}_{k}^{\left(2\right)}$ can be used. From standard theory for OLS estimators, the joint distribution of the ${\hat{\beta }}_{k}^{\left(1\right)}$ is as follows

$$({\hat{\beta }}_{1}^{\left(1\right)},\ldots ,{\hat{\beta }}_{K}^{\left(1\right)}{)}^{T}∼MVN\left((\beta_{1},\ldots ,\beta_{k}{)}^{T},\;\sigma^{2}{\left[D^{(1)T}D^{\left(1\right)}\right]}^{-1}\right)$$

where $D^{\left(1\right)}$ denotes the observed design matrix for the model fitted to the stage one data (which includes any covariate information). Meanwhile, the marginal distribution for ${\hat{\beta }}_{s}^{\left(2\right)}$ , corresponding to a treatment s selected to continue to stage two is given by

$${\hat{\beta }}_{s}^{\left(2\right)}∼N(\beta_{s},\;\sigma^{2}{\left[D^{(2)T}D^{\left(2\right)}\right]}_{s,s}^{-1})$$

where $D^{\left(2\right)}$ denotes the observed design matrix for the model fitted to the stage two data. Using these distributional properties, we can use the theory given in ^53^ to derive the Uniformly Minimum Variance Conditionally Unbiased Estimator (UMVCUE). As the name suggests, this estimator has the minimum variance out of the class of unbiased estimators. It is conditionally unbiased since we condition on the (pre-defined) selection rule used in the trial.

The UMVCUE for a treatment effect $\beta_{s}$ given some selection rule Q is as follows:

(2) $${\hat{U}}_{s}=\frac{\int_{A}\frac{y_{s}}{\sqrt{2\pi \eta_{s}^{2}}}\exp\;\left[-\frac{1}{2\eta_{s}^{2}}{\left(y_{s}-\frac{\tau_{(2),s}^{2}Z_{s}}{\tau_{(1),s}^{2}+\tau_{(2),s}^{2}}\right)}^{2}\right]dy_{s}}{\int_{A}\frac{1}{\sqrt{2\pi \eta_{s}^{2}}}\exp\;\left[-\frac{1}{2\eta_{s}^{2}}{\left(y_{s}-\frac{\tau_{(2),s}^{2}Z_{s}}{\tau_{(1),s}^{2}+\tau_{(2),s}^{2}}\right)}^{2}\right]dy_{s}}$$

where

$$\begin{array}{rl} \eta_{s} & =\frac{\tau_{(2),s}^{2}}{\sqrt{\tau_{(1),s}^{2}+\tau_{(2),s}^{2}}},\;Z_{s}={\hat{\beta }}_{s}^{\left(1\right)}+\frac{\tau_{(1),s}^{2}}{\tau_{(2),s}^{2}}{\hat{\beta }}_{s}^{\left(2\right)},\\ \tau_{(1),s}^{2} & =\sigma^{2}{\left[D^{(1)T}D^{\left(1\right)}\right]}_{s,s}^{-1},\;\tau_{(2),s}^{2}=\sigma^{2}{\left[D^{(2)T}D^{\left(2\right)}\right]}_{s,s}^{-1} \end{array}$$

and $\int_{A}$ denotes integrating over the possible values of ${\hat{\beta }}_{s}^{\left(2\right)}$ given the the selection rule Q and $\left(z_{1},\ldots ,z_{K}\right)$ .

As an explicit example, consider selection rules based on ranking the treatments by their z -statistics, where (without loss of generality) the treatments are relabelled so that this corresponds to the event

$$Q=\left\{\frac{{\hat{\beta }}_{1}^{\left(1\right)}}{\tau_{(1),1}}\geq \frac{{\hat{\beta }}_{2}^{\left(1\right)}}{\tau_{(1),2}}\geq \cdots \geq \frac{{\hat{\beta }}_{K}^{\left(1\right)}}{\tau_{(1),K}}\right\}.$$

A common selection rule would then be to select the top k treatments based on this ranking (where k is pre-defined). Using this selection rule, the UMVCUE for $\beta_{s}$ given Q has the following closed form expression:

(3) $${\hat{U}}_{s}=\frac{\tau_{(2),s}^{2}Z_{s,s}}{\tau_{(1),s}^{2}+\tau_{(2),s}^{2}}-\frac{\tau_{(2),s}^{2}}{\sqrt{\tau_{(1),s}^{2}+\tau_{(2),s}^{2}}}\frac{\phi (W_{1})-\phi (W_{2})}{\Phi (W_{1})-\Phi (W_{2})}$$

where

$$\begin{array}{rl} & Z_{i,s}={\hat{\beta }}_{i}^{\left(1\right)}+\frac{V_{i,s}}{\tau_{(2),s}^{2}}{\hat{\beta }}_{1}^{\left(2\right)}\;\text{\textbackslash{},for }\;i=1,\ldots ,K\\ & W_{l}=\frac{c_{j}\sqrt{\tau_{(1),s}^{2}+\tau_{(2),s}^{2}}}{\tau_{(2),s}^{2}}-\frac{Z_{s,s}}{\sqrt{\tau_{(1),s}^{2}+\tau_{(2),s}^{2}}}\;\text{\textbackslash{},for }\;l=1,2\\ & c_{1}=\min\left\{\frac{\tau_{(2),s}^{2}\;\left[\tau_{(1),i+1}\;Z_{i,s}-\tau_{(1),i}\;Z_{i+1,s}\right]}{\tau_{(1),i+1}\;V_{i,s}-\tau_{(1),i}\;V_{i+1,s}}\;:\;\tau_{(1),i+1}\;V_{i,s}>\tau_{(1),i}\;V_{i+1,s};i=1,\ldots ,K-1\right\},\\ & c_{2}=\max\left\{\frac{\tau_{(2),s}^{2}\;\left[\tau_{(1),i+1}\;Z_{i,s}-\tau_{(1),i}\;Z_{i+1,s}\right]}{\tau_{(1),i+1}\;V_{i,s}-\tau_{(1),i}\;V_{i+1,s}}\;:\;\tau_{(1),i+1}\;V_{i,s}<\tau_{(1),i}\;V_{i+1,s};i=1,\ldots ,K-1\right\},\\ & V_{i,s}=\sigma^{2}{\left[D^{(1)T}D^{\left(1\right)}\right]}_{i,s}^{-1} \end{array}$$

Note that for $c_{1}$ and $c_{2}$ , we define min{∅}=+∞ and max{∅}=−∞ , where ∅ denotes the empty set. When $c_{1}=\infty$ this corresponds to the selection rule not inducing an upper limit to the stage 2 estimator (considered as a function of the complete sufficient statistic), and similarly $c_{2}=-\infty$ corresponds to the selection rule not inducing a lower limit.

## 4. Illustration of AMADs with normal endpoint and covariates

Following the above example of a selection rule where the seemingly best intervention is selected at interim analysis, we explore the impact of covariate adjustment on several aspects of an AMAD with a simulation study. Note that the total number of patients in this two-stage AMAD is J×(K+1)+2J , where each study arm has the same number of patients per stage.

We describe the background of our simulation study and some performance measures. We look at the simulation results that cover the treatment selection characteristics of AMADs and the analysis perspective when different sets of covariates are considered in the modelling approach. For each simulation scenario, which consists of a data generating mechanism (DGM) under a specific parameter configuration for the treatment effects, we conducted 100,000 trial replications using R version 4.0.5.^54^ An example R script is available in the supplemental material.

### 4.1. Simulation settings and performance measures

Consider a simple example where there is a total of K=2 active interventions, which are denoted T1 and T2 respectively. Consider the presence of two independent normally distributed covariates and a normal outcome. The following is the joint distribution of the outcome and the covariates for each arm k=0,T1,T2 :

$$\begin{array}{r} \left(\begin{array}{c} X1_{\;jk}^{\left(t\right)}\\ X2_{\;jk}^{\left(t\right)}\\ Y_{\;jk}^{\left(t\right)} \end{array}\right)∼MVN\left(\left[\begin{array}{c} \mu_{k,X1}\\ \mu_{k,X2}\\ \mu_{k,Y} \end{array}\right],\left[\begin{array}{ccc} \sigma_{k,X1}^{2} & 0 & \rho_{k,X1,Y}\sigma_{k,X1}\sigma_{k,Y}\\ 0 & \sigma_{k,X2}^{2} & \rho_{k,X2,Y}\sigma_{k,X2}\sigma_{k,Y}\\ \rho_{k,X1,Y}\sigma_{k,X1}\sigma_{k,Y} & \rho_{k,X2,Y}\sigma_{k,X2}\sigma_{k,Y} & \sigma_{k,Y}^{2} \end{array}\right]\right), \end{array}$$

where $\mu_{k,v}$ and $\sigma_{k,v}^{2}$ are the mean and variance of variable $v=X1_{\;jk}^{\left(t\right)},X2_{\;jk}^{\left(t\right)},Y_{\;jk}^{\left(t\right)},$ and $\rho_{k}=\{\rho_{k,X1,Y},\rho_{k,X2,Y}\}$ denotes the correlations between the covariates and the outcome variable. Given a DGM with a specific parameter configuration for the treatment effects, we simulate responses and the covariates from this multivariate normal distribution.

In terms of the treatment selection rule used, we assume that one intervention s is selected, where (using the notation from Section 3.1.)

$$s={\text{argmax}}_{k\in \{T1,T2\}}\left\{\frac{{\hat{\beta }}_{k}^{\left(1\right)}}{\tau_{(1),k}}\right\}$$

i.e. the intervention with the largest stage one z -statistic is selected. In practice, σ is unknown. So in our simulations we use t -statistics rather than z -statistics for both the treatment selection and the final inference. The difference will be minimal except in small samples.

In the simulation, we set $\mu_{k,\;X1}=\mu_{k,\;X2}=0.5$ and $\sigma_{k,v}^{2}=1\;\forall k,\;v$ . Note that the values of $\mu_{k,\;X1}$ and $\mu_{k,\;X2}$ have no impact on the performance measures as we simulate the covariates and responses from their joint distribution.

#### 4.1.1. Data generating mechanisms and parameter configuration for treatment effects

Each of the following DGMs are considered in our simulation study:

- DGM1: no correlation between the covariates and the outcome, i.e., $\rho_{k}=\{0,0\}$ ∀k
- DGM2: $X1_{k}$ is not correlated with $Y_{k}$ , but $X2_{k}$ is correlated weakly with $Y_{k}$ ∀k , i.e., $\rho_{k}=\{0,0.2\}$
- DGM3: both $X1_{k}$ and $X2_{k}$ are correlated with $Y_{k}$ weakly ∀k , i.e., $\rho_{k}=\{0.2,0.2\}$
- DGM4: $X1_{k}$ is correlated with $Y_{k}$ weakly, but $X2_{k}$ is correlated with $Y_{k}$ moderately ∀k , i.e., $\rho_{k}=\{0.1,0.6\}$

For each DGM, we run simulations for scenarios that are defined by the following parameter configuration for $\mu_{Y}=\{\mu_{0,Y},\mu_{1,Y},\mu_{2,Y}\}$ :

- Null: the global null configuration that has $\mu_{Y}=\{0,0,0\}$
- LFC50: a least-favourable-configuration that has $\mu_{Y}=\{0,0,0.22\}$
- LFC80: a least-favourable-configuration that has $\mu_{Y}=\{0,0,0.304\}$
- STEP50: a stepwise-configuration that has $\mu_{Y}=\{0,0.11,0.22\}$
- STEP80: a stepwise-configuration that has $\mu_{Y}=\{0,0.152,0.304\}$

The above numerical examples of effect sizes have been chosen as follows. Since we want to explore the impact of covariate adjustment on the operating characteristics of an AMAD, we fix the total sample size and identify the effect sizes required for a level of power under an alternative hypothesis. More specifically, consider J=100 for each arm per each stage, the value of $\mu_{2,Y}$ is chosen to achieve a 50 % and 80 % power to reject any one (out of the two) hypothesis under DGM1 with the LFC50 and LFC80 parameter configurations respectively, when the unadjusted approach is implemented, i.e., without covariate adjustment. The inverse normal combination test is used to control FWER in the strong sense at a significance level of 2.5%. For illustration purpose, we consider similar values of $\mu_{2,Y}$ and let $\mu_{1,Y}=0.5\mu_{2,Y}$ for the stepwise scenarios.

#### 4.1.2. Comparators: modelling approaches

Since there are two covariates considered in the simulation study, we consider the following modelling approaches for both the interim and final analyses, using the linear model defined by equation (1) in Section 3.1.:

- Unadjusted: a linear model for the outcome on the treatment variable only, ignoring the covariate information, i.e., setting $\gamma_{1}=\gamma_{2}=0$ in equation (1). This is similar to considering the difference between the average mean responses of the comparator groups.
- Adjusted ${}_{X1}$ : a linear model for the outcome on the treatment variable and $X1_{\;jk}$ only, i.e., setting $\gamma_{2}=0$ in equation (1).
- Adjusted ${}_{X2}$ : a linear model for the outcome on the treatment variable and $X2_{\;jk}$ only, i.e., setting $\gamma_{1}=0$ in equation (1).
- Adjusted ${}_{both}$ : a linear model for the outcome on the treatment variable, $X1_{\;jk}$ and $X2_{\;jk}$ .

#### 4.1.3. Performance measures

To illustrate the role of covariate adjustment in AMADs, we examine the following performance measures to make comparisons between the analysis approaches under different scenarios in the simulation study.

Regarding the treatment selection operating characteristics of the AMAD, we consider the frequency when one specific treatment is selected over trial replications and the frequency when an adjusted approach selects a different treatment to the unadjusted approach. The former reflects if the AMAD is selecting the truly effective arm(s) correctly based on the interim analysis; a higher frequency of selecting the truly effective arm(s) is desirable. The latter reflects how often an adjusted modelling approach could lead to a different treatment selection outcome to the unadjusted analysis approach. This indicates the disparity of the treatment selection outcome when covariate adjustment is considered in the framework of an AMAD.

For the inferential properties of the AMAD, we evaluate the probability of rejecting (any) one hypothesis and the marginal probability of rejecting a specific hypothesis under the above described scenarios. We look at the standard properties of different estimators following different modelling approaches in terms of the bias and mean squared error (MSE). In what follows, the bias of an estimator $\hat{\beta }$ for a parameter β is defined as $E[\hat{\beta }]-\beta$ , while the MSE is defined as $E[(\hat{\beta }-\beta {)}^{2}]$ .

### 4.2. Simulation results

We describe the simulation results by part: i) Frequency of selecting a specific treatment, ii) Probability of rejecting a hypothesis, and iii) Properties of different estimators of the treatment effect.

#### 4.2.1. Frequency of selecting a specific treatment

First consider the treatment selection operating characteristics of the AMAD. For the LFC parameter configurations, it is desirable to select T2 as frequently as possible. When the interim data is such that T1 is selected, that means an incorrect selection has been made and the study will fail to detect a truly effective treatment. For the stepwise-configurations, selecting T1 is not an incorrect selection but it is less desirable than selecting T2 as T2 is more effective than T1. For the null scenario where the interventions have the same effectiveness to the control treatment, it does not matter which intervention is selected when the DGMs have such a parameter configurations. For this reason we exclude the null scenario in this part of the results.

**Figure 1.**
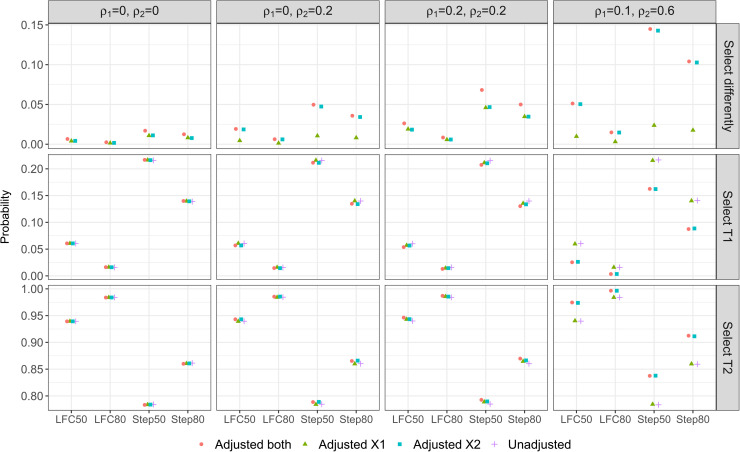
Treatment selection frequency/ probability: when an adjusted approach has a different selection result to the unadjusted approach (first panel), when both an adjusted approach and the unadjusted approach select T1 (second panel), and when both an adjusted approach and the unadjusted approach select T2 (third panel).

Figure 1 shows the result from our simulation study under some DGMs (column wise) with different parameter configurations for the mean responses (x-axis). The first row of plots shows that when adjusting for correlated covariates, the result of treatment selection can be different to that from the unadjusted approach; this can be as high as 14% of the times when DGM4 with STEP50 is the true scenario, i.e., see Adjusted ${}_{both}$ and Adjusted ${}_{X2}$ in the first plot of column four in Figure 1. When covariates are not correlated with the outcome variable, as in DGM1, this occurrence is less frequent when comparing an adjusted approach with an unadjusted approach; the probability is less than 2.5% for all the comparisons. This observation highlights the importance of specifying which covariates to adjust for when performing treatment selection at interim analyses, since adjusting for different sets of covariate can lead to different treatment selection outcome given the data of one trial replication.

The second and third row of plots shows the probability that T1 and T2 are selected, respectively, to continue with the control treatment to the second stage of the study. (The third row is the complement of the second row as probability sums to one.) In general, we find that adjusting for covariates that are truly correlated with the outcome can reduce the probability that T1 is selected and increase the probability that T2 is selected under all the considered non-null scenarios, from the probabilities that are obtained by the unadjusted approach. This is desirable as T2 is the most effective treatment among the three options across the scenarios on the x-axis of Figure 1. See for example Step 80 in the third column of plots for DGM3 where Adjusted ${}_{both}$ is the desirable approach.

Moreover, we find that adjusting for a highly correlated covariate can lead to a better treatment selection result even when the weakly correlated covariate has been omitted. This is reflected by Adjusted ${}_{X2}$ in the plots in column four of Figure 1 where Adjusted ${}_{both}$ is the desirable approach for DGM 4.

When the correlations of the covariates are weak, failing to include the covariates in the modelling approaches has little impact on the probability of treatment selection when compared with the desirable approach. This is observed from the unadjusted approach and Adjusted ${}_{X1}$ in the second column of plots where Adjusted ${}_{X2}$ is the desirable approach for DGM2; and the unadjusted approach, Adjusted ${}_{X1}$ and Adjusted ${}_{X2}$ in the third column of plots where Adjusted ${}_{both}$ is the desirable approach for DGM3. Nevertheless the disparity between the probabilities is tiny when comparing across the analysis approaches for a given parameter configuration under these DGMs; it is more noticeable for the stepwise-configurations than for the LFC configurations.

On the other hand when uncorrelated covariates are mistakenly being included in the model, the probability of one treatment being selected is similar to the approach that has the correct adjustment. For example, the desirable approach for DGM1 is the unadjusted approach, both the plots in column one of Figure 1 show that all the other three approaches have similar performance to the unadjusted approach. Similar finding is observed for Adjusted ${}_{both}$ in the second column of plots where Adjusted ${}_{X2}$ is the desirable approach for DGM2.

In summary, adjusting for covariates that are highly correlated with the outcome leads to a higher chance of selecting the truly best treatment in AMADs, whereas including uncorrelated covariates or omitting weakly correlated covariates has little impact on the selection result when compared with the unadjusted approach. The maximum and minimum of the probabilities of treatment selection and their differences are presented in Table S1 in the supplemental material.

#### 4.2.2. Probability of rejecting a hypothesis

We focus on the results of hypothesis tests for AMADs. Recall that we have two elementary null hypotheses, $H_{01}\;:\;\mu_{1,Y}=0$ , and $H_{02}\;:\;\mu_{2,Y}=0$ . Since the effect sizes of the illustrations are chosen with the unadjusted approach under DGM 1 based on the LFC settings and the power is defined by the probability of detecting any one effective treatment, the marginal probability of rejecting $H_{01}$ and $H_{02}$ respectively can vary according to the non-null parameter configurations and be affected by covariate adjustment.

Table 1 shows the probability of hypothesis rejections under the null scenario for each combination of DGM and the analysis approaches. Overall we find that covariate adjustment has negligible impact on FWER if there is any: we observed values that are close to 2.5% from our simulation across the analysis approaches, and they lie within the expected 95% confidence interval of the true FWER. The marginal probability of rejecting $H_{01}$ and the marginal probability of rejecting $H_{02}$ are both close to 1.3%.

**Table 1.** Probability of hypothesis rejections under the null scenario for each DGM when each analysis approach is implemented. The Monte-Carlo simulation error for the nominal FWER is $\sqrt{0.025*0.975/100000}$ and the 95% confidence interval for the nominal FWER is [0.02403,0.02597] .

	$\rho_{1}=0$	$\rho_{1}=0$	$\rho_{1}=0.2$	$\rho_{1}=0.1$
	$\rho_{2}=0$	$\rho_{2}=0.2$	$\rho_{2}=0.2$	$\rho_{2}=0.6$
Approach	FWER		
Unadjusted	0.02520	0.02522	0.02564	0.02574
Adjusted ${}_{X1}$	0.02527	0.02551	0.02501	0.02536
Adjusted ${}_{X2}$	0.02530	0.02542	0.02569	0.02541
Adjusted ${}_{both}$	0.02534	0.02530	0.02488	0.02495
	Probability of rejecting $H_{01}$		
Unadjusted	0.01272	0.01243	0.01271	0.01274
Adjusted ${}_{X1}$	0.01257	0.01253	0.01254	0.01273
Adjusted ${}_{X2}$	0.01265	0.01294	0.01278	0.01305
Adjusted ${}_{both}$	0.01272	0.01274	0.01266	0.01281
	Probability of rejecting $H_{02}$		
Unadjusted	0.01248	0.01279	0.01293	0.01300
Adjusted ${}_{X1}$	0.01270	0.01298	0.01247	0.01263
Adjusted ${}_{X2}$	0.01265	0.01248	0.01291	0.01236
Adjusted ${}_{both}$	0.01262	0.01256	0.01222	0.01214

**Figure 2.**
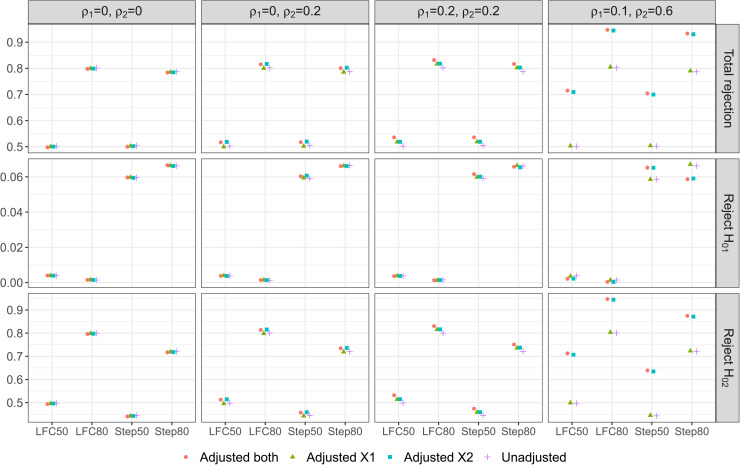
Unconditional rejection probabilities from the two-stage AMAD: when any one hypothesis is rejected (first panel), when $H_{01}$ is rejected (second panel), and when $H_{02}$ is rejected (third panel).

For the non-null scanarios, Figure 2 shows the unconditional probabilities of hypothesis rejections from our simulation study. First consider the probability of rejecting any one of the hypotheses, i.e., the overall power under the non-null scenarios. We find that accounting for uncorrelated covariates in the final analysis has a negligible impact on the overall power when compared with the unadjusted approach. For example all three adjustment approaches have similar level of power to that of the unadjusted method in the first plot of column one under DGM 1; Adjusted ${}_{X1}$ has a similar performance to the unadjusted approach under DGM 2 where X1 is not correlated with the outcome variable.

When adjusting for at least one covariate that is correlated with the outcome, we find that the overall power becomes higher than that achieved by the unadjusted approach. This is true even when an approach includes an uncorrelated covariate (to the outcome) in addition to the covariate that is correlated with the outcome; for example, see the first plot in column two of Figure 2 where Adjusted ${}_{both}$ gives a probability that is of similar magnitude to that obtained from the desirable approach, Adjusted ${}_{X2}$ , where X1 is not correlated with the outcome variable under DGM 2.

This highlights the benefit of covariate adjustments on the overall power of AMAD. The increases in the overall power can be as high as 21% under DGM 4 when either Adjusted ${}_{X2}$ or Adjusted ${}_{both}$ is implemented for scenarios where the parameter configurations follow LFC50 and Step50 respectively.

Now focus on the probability of rejecting $H_{01}$ , i.e., the second row of plots in Figure 2. For LFC50 and LFC80, this marginal probability can be considered as a type I error rate because T1 has the same effect as the control treatment. We see that under DGMs 1-3 with these parameter configurations, all the analysis approaches lead to a similar level to this probability; under DGM 4, Adjusted ${}_{X1}$ and the unadjusted approach give a slightly higher rate than the other two approaches that adjust for X2, which is a covariate that is highly correlated with the outcome. This observation may be related to the frequency of selecting T1 at interim analysis: in Figure 1 second row of plots, all the four approaches select T1 at a similar level of frequency under DGMs 1-3, whereas there are some noticeable differences in the frequency level under DGM 4 for LFC50 and LFC80. In the later case, Adjusted ${}_{X1}$ and the unadjusted approach select T1 more frequently than the other two approaches, which may lead to a higher rejection frequency on average.

For Step50 and Step80, the probability of rejecting $H_{01}$ can be considered as a marginal power because T1 is more effective than the control treatment but it is less effective than T2. Similar to the other two parameter configurations, probability of rejecting $H_{01}$ is fairly consistent across the four analysis approaches under DGMs 1-3. But, under DGM 4, Adjusted ${}_{X1}$ and the unadjusted approach give a lower marginal power than the other two approaches for scenario Step50 but the opposite for scenario Step80. This is an interesting observation and might be explained as follows. From Figure 1 second row fourth column, we see that Adjusted ${}_{X1}$ and the unadjusted approach select T1 more frequently than the other two approaches for DGM 4 with both Step50 and Step80. When the effect size to detect is small as in scenario Step 50, failing to account for the covariate that is highly correlated to the outcome in the final analysis leads to a loss in the marginal power of rejecting $H_{01}$ . On the other hand for Step80, the other two approaches have a lower marginal power than Adjusted ${}_{X1}$ and the unadjusted approach, which is likely due to their lower frequency in selecting T1.

Lastly for the marginal probability of rejecting $H_{02}$ , the same observations for the overall power applies: adjusting for covariates that are correlated with the outcome improves the power over the unadjusted approach. The gain in power by covariate adjustment is larger when the effect size is small than when effect size is large; this is due to the non-linear relationship between the power and the effect size. On the other hand, including covariates that are not correlated with the outcome in the analysis unknowingly has minimal impact on the power when compared with the unadjusted approach.

#### 4.2.3. Treatment effect estimators

**Figure 3.**
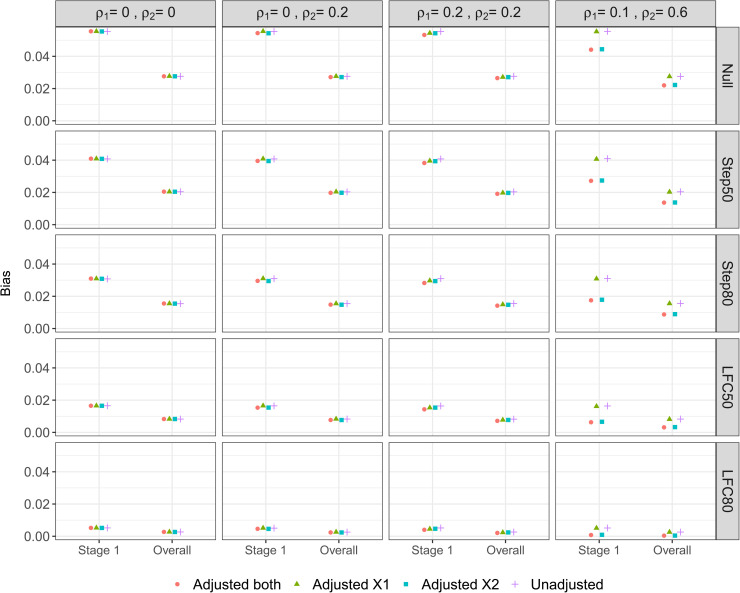
Bias of the Stage one and Overall estimators of the treatment effect.

We consider the properties of the treatment effect estimators described in Section 3.2.. First we focus on the bias and then the MSE of these estimators, as defined in Section 4.1.3.. Figure 3 shows the bias of the stage one and Overall estimators of the treatment effect under the different scenarios for $\mu_{Y}$ . We do not show the bias for the stage two estimator and UMVCUE, since both are theoretically unbiased under all scenarios and adjustment methods, which was borne out in the simulation results. Note that the UMVCUE was empirically unbiased even though it was derived under the assumption of known values of $\tau_{(1),k}$ , whereas in the simulations we estimate this from the trial data.

Looking across the different scenarios, both the Stage one and Overall estimators exhibit a positive bias and the magnitude of this bias has the following ordering (for any given adjustment method): Null > Step50 > Step80 > LFC50 > LFC80. This reflects how the upwards selection pressure is greatest for the Null scenario (since all the treatment effects are identical), which then decreases as the treatment effects become more distinct, i.e. as the difference $(\mu_{2,Y}-\mu_{0,Y})-(\mu_{1,Y}-\mu_{0,Y})=\mu_{2,Y}-\mu_{1,Y}$ increases. The magnitude of the bias can be relatively substantial – for example, under DGM 1 with the Step50 scenario, the bias of the Stage one and Overall estimators is 0.04 and 0.02 respectively, while $\mu_{Y}=(0,0.11,0.22)$ . For any given adjustment approaches, the bias of the Overall estimator is approximately 50% that of the bias of the Stage one estimator. This is because the Overall estimator combines the biased Stage one estimator and unbiased Stage two estimator, with the same number of patients per arm for Stages one and two.

Looking now at the different adjustment approaches, when the correlation between the covariates and outcome is zero or low (i.e. under DGMs 1-3), there is minimal difference in the bias of either the Stage one or Overall estimators. In particular, there is no loss in terms of increased bias when adjusting for covariates that are uncorrelated with the outcome (i.e. DGM 1). When there is a moderate correlation between covariate X2 and the outcome (DGM 4), there is a noticeable decrease in the bias when adjusting for X2 (i.e. Adjusted ${}_{X2}$ and Adjusted ${}_{both}$ ). For example, under the Step50 scenario the bias of the Overall estimator decreases by 33% when comparing the Unadjusted analysis with Adjusted ${}_{both}$ .

**Figure 4.**
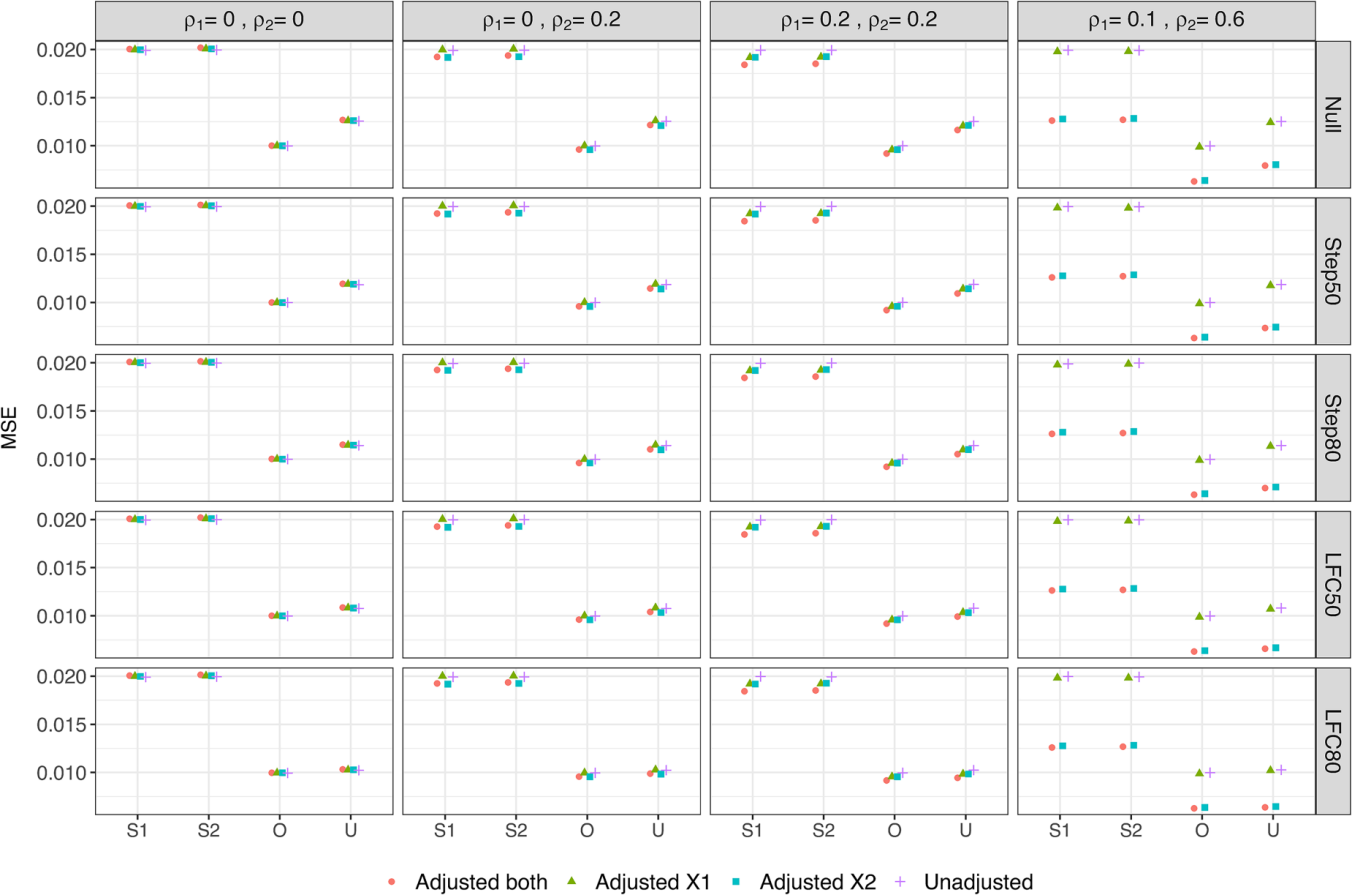
Mean Square Error of various estimators of the treatment effect. S1 = Stage one, S2 = Stage two, O = Overall, U = UMVCUE.

Figure 4 shows the MSE of the Stage one, Stage two and Overall estimators as well as the UMVCUE. Note that since the Stage two estimator and the UMVCUE are unbiased, the MSE is equal to the mean variance of these estimators. For any given adjustment approach, the MSE of the Stage one and Stage two estimators are virtually identical, and double the MSE of the Overall estimator. As well, the MSE of the Stage one, Stage two and Overall estimators remain virtually identical across the different scenarios for $\mu_{Y}$ .

For the UMVCUE, the MSE has the same ordering (for any given adjustment approach) as the ordering for the bias of the Stage one and Overall estimators, i.e. Null > Step50 > Step80 > LFC50 > LFC80. This reflects how the larger adjustments made by the UMVUCE to the Overall estimator in scenarios with a larger bias results in a larger variance and hence MSE. The UMVCUE has a larger MSE than the Overall estimator (up to a maximum increase of 25%, under the null scenario), with the magnitude of this difference having the same ordering as above. Hence the UMVCUE pays the largest price in terms of MSE precisely in the scenarios where the Overall estimator pays the largest price in terms of bias. Reassuringly, when there is little bias in the Overall estimator (e.g. in the LFC80 scenario) then the MSE of the Overall estimator and UMVCUE are virtually identical.

Finally, looking at the different adjustment approaches, when there is no or low correlation between the covariates and outcome, there is little difference in the MSE for a given estimator. In particular, there is no increase in MSE when adjusting for covariates that are uncorrelated with the outcome. When there is a moderate correlation between covariate X2 and the outcome (DGM 4), we observe a noticeable decrease in the MSE when adjusting for X2. For example, the MSE of the Overall estimator decreases by 39% when comparing the Unadjusted analysis with Adjusted ${}_{both}$ .

### 4.3. Case illustration: INHANCE study

One of the objectives of the INHANCE study was to demonstrate the efficacy of indacaterol (intervention) versus placebo on patients with moderate-to-severe chronic obstructive pulmonary disease.^43^ An adaptive seamless phase IIb/ IIIa study design was used to screen six interventions at stage one (with 376 patients randomized to seven arms including the placebo group), and select three interventions to be continued with the placebo group to stage two based on 2-week efficacy and safety data. A total of 1683 patients were randomised to four arms at the second stage of the study: placebo, double-blind indacaterol 150 μ g, double-blind indacaterol 300 μ g or open-label tiotropium 18 μ g. The primary efficacy endpoint was forced expiratory volume (FEV) measured at 24 hours post dose after 12 weeks. A Bonferroni correction was applied to account for testing multiple intervention versus placebo comparisons, and a mixed model analysis of covariance was employed in the intention-to-treat analysis, with treatment, smoking status and country as fixed effects and baseline FEV and baseline reversibility as covariates, and centre within country as random intercept. The covariate adjusted result based on stage two data indicated that all the three interventions are more efficacious than the placebo.

To illustrate what might happen using the AMAD framework, consider a hypothetical scenario where the placebo and three interventions mentioned above are to be evaluated again using a similar design, but with only two of them selected to continue (together with the placebo) in the subsequent stage. This can be considered as a confirmatory two-stage multi-arm design where a strict control of the FWER is required when one intervention arm is closed at the end of stage one. Here we focus on using the treatment selection rule where the best two interventions would be selected, as opposed to using some pre-defined progression criteria based on the safety and efficacy data. We use the observed efficacy and covariate information (in terms of the means and standard deviations) from the INHANCE study, as given in Table 2 to simulate a realisation of this trial design. For all interventions, the assumed standard deviations of the efficacy endpoint (FEV), baseline FEV and reversibility were 0.375 L, 0.504 L and 16.63%, respectively. For illustration, we used a sample size of J=100 subjects per arm per stage to simulate one replication of the hypothetical study.

**Table 2.** Observed results from the INHANCE study.

**Intervention**	**Efficacy**	**Covariate**	
	Mean FEV (L)	Baseline FEV (L)	Reversibility (%)
0: Placebo	1.28	1.52	15.6
1: Indacaterol (150 μ g)	1.46	1.53	15.2
2: Indacaterol (300 μ g)	1.46	1.45	15.6
3: Tiotropium	1.42	1.51	15.5

Table 3 shows the selected interventions and rejected hypotheses under different adjustment methods. We see that under Unadjusted, Adjusted ${}_{X1}$ and Adjusted ${}_{X2}$ , the same interventions (1 and 3) are selected, whereas under Adjusted ${}_{both}$ , interventions 2 and 3 are selected. The corresponding null hypotheses $H_{0k}$ (that the treatment difference for intervention k is equal to zero) for the selected treatments are all rejected under the adjustment methods, except for the unadjusted analysis where interventions 1 and 3 are selected but only hypothesis $H_{03}$ can be rejected.

**Table 3.** Selected interventions and rejected hypotheses under different adjusted methods.

**Adjustment**	**Selections**	**Rejections**
Unadjusted	1, 3	$H_{03}$
Adjusted ${}_{X1}$	1, 3	$H_{01},H_{03}$
Adjusted ${}_{X2}$	1, 3	$H_{01},H_{03}$
Adjusted ${}_{both}$	2, 3	$H_{02},H_{03}$

Table 4 gives the values of the various estimators (Stage one, Stage two, Overall and UMVCUE) as well as the standard confidence intervals (Stage one, Stage two, Overall) for the treatment difference of the two selected interventions for each adjustment method. We see that the Overall estimate for intervention 3 is very similar regardless of the adjustment method used. In contrast, the Stage one estimate and UMVCUE are noticeably larger under Unadjusted and Adjusted ${}_{X2}$ compared to under Adjusted ${}_{X1}$ and Adjusted ${}_{both}$ , with the opposite seen for the Stage two estimate. As for intervention 1, the Overall and Stage two estimates have the following ordering: Adjusted ${}_{X2}$ > Adjusted ${}_{X1}$ > Unadjusted. The UMVUCE has virtually identical estimates under Adjusted ${}_{X1}$ and Adjusted ${}_{X2}$ , which are both noticeably larger than the UMVUCE under an Unadjusted analysis.

**Table 4.** Estimates (and standard confidence intervals) for the treatment difference with placebo for the selected interventions under different adjustment methods.

		**Estimates**			
**Adjustment**		**Stage one**	**Stage two**	**Overall**	**UMVCUE**
Unadjusted	1	0.115 (0.02, 0.21)	0.005 ( −0.10 , 0.11)	0.060 ( −0.01 , 0.13)	0.015
	3	0.193 (0.09, 0.29)	0.033 ( −0.07 , 0.14)	0.113 (0.04, 0.18)	0.116
Adjusted ${}_{X1}$	1	0.129 (0.04, 0.21)	0.044 ( −0.05 , 0.14)	0.086 (0.04, 0.21)	0.050
	3	0.166 (0.08, 0.25)	0.064 ( −0.03 , 0.16)	0.115 (0.05, 0.18)	0.107
Adjusted ${}_{X2}$	1	0.126 (0.04, 0.21)	0.063 ( −0.03 , 0.15)	0.095 (0.03, 0.15)	0.050
	3	0.192 (0.11, 0.27)	0.036 ( −0.05 , 0.12)	0.114 (0.05, 0.17)	0.117
Adjusted ${}_{both}$	2	0.141 (0.07, 0.21)	0.184 (0.12, 0.25)	0.164 (0.12, 0.21)	0.162
	3	0.164 (0.10, 0.23)	0.066 ( −0.00 , 0.13)	0.116 (0.07, 0.16)	0.095

Across all the adjustment methods, the Stage one estimate is substantially larger than the Stage two estimate for interventions 1 and 3, which is particularly noticeable for intervention 3. This reflects the upward selection pressure on the top and second-ranked interventions in Stage one. For interventions 1 and 3, the Overall estimate is approximately halfway between the Stage one and Stage two estimates, while the UMVCUE corrects the Overall estimate towards the unbiased Stage two estimate. Interestingly, this correction is minimal when not adjusting for X1 (i.e under an Unadjusted and Adjusted ${}_{X2}$ ). Finally, for intervention 2, which is only selected under Adjusted ${}_{both}$ , the Stage two estimate actually increases from Stage one, and the Overall estimate and UMVCUE are virtually identical in this case.

## 5. Marginal vs conditional measures: binary and survival outcomes

For a continuous outcome where the treatment effect measures are normally distributed, we have illustrated that adjusting for covariates can improve the trial operating characteristics and the precision of the estimated treatment effects. For binary and survival outcomes, covariate adjustment requires extra considerations when the main interest is usually in the odds ratio and the hazard ratio. The problem arises because the odds ratio and the hazard ratio are non-collapsible, meaning that the conditional estimand is not equivalent to the marginal estimand of these treatment effect measures.^55^ In this section, we briefly revisit the concept of non-collapsibility and highlight the role of covariate adjustment in the context of AMADs.

Recall that a conditional odds ratio (or hazard ratio) describes the effect on the outcome of an individual patient when taking an intervention instead of the control treatment; the marginal odds ratio (or hazard ratio) describes the effect on the outcome of the samples of a target population when they were given the intervention instead of the control treatment. The conditional estimand accounts for the individual patient characteristics or background via the incorporation of the associated covariates in the regression models. On the other hand, the marginal estimand depends on the distribution of the associated covariates implicitly, meaning that the estimate of a marginal odds ratio (or hazard ratio) obtained from a study may not be applicable to other populations that have different characteristics, see for example the illustration in Figure 1 of Groenwold et al. ^56^.

The subtlety in the interpretation of the marginal versus conditional odds ratio (or hazard ratio) make them non-comparable with respect to the benefits of covariate adjustment. This is not the case for continuous outcome where the conditional and the marginal estimand are equivalent in nature (but not numerically as obtained from different regression models). Other measures that are collapsible include the absolute risk difference, the relative risk difference, the log odds ratio and the hazard difference in the additive hazards models. The interested reader is referred to Daniel et al. ^55^ and the references therein for more details about non-collapsibility.

We emphasise that when the odds ratio (or hazard ratio) is used as a measure of treatment effect in a trial, the pre-specification in the statistical analysis plan of which covariates to be included in the analyses (both interim and final) is paramount. One should not make comparisons of treatment effect estimates from analyses with different sets of covariates because the resulting conditional treatment effects convey different information. In the context of AMADs, the same set of covariates should be included in both the treatment selection and the final analyses for the same reason.

The consideration of marginal odds ratio (or hazard ratio) is less useful since the focus of early phase clinical studies often is on the average treatment effect on individual patients, and not the average treatment benefits for the target population. For AMADs, a caveat to using the marginal odds ratio or the marginal hazard ratio for both the treatment selection and the final analyses is that the patient composition may vary across stages. Consequently the stage wise parameter estimates may not be congruent in terms of the applicability of the results to the target population when the distribution of the characteristics of patients recruited in stage one is different to that in stage two.

Nevertheless, one may produce a marginalised-covariate-adjusted odds ratio (or hazard ratio) as supplemental information following the procedure in Daniel et al. ^55^. However, it might be less straightforward to produce the UMVCUE for these treatment effect measures using the procedure as the standard deviation of the stage-wise parameters requires approximation by using either the Delta method or bootstrapping.

## 6. Discussion

Within a two-stage design, we have evaluated the impact of covariate adjustments on the operating characteristics of AMADs and the properties of treatment effect estimators. More simulation results on trial operating characteristics in the presence of additional six covariates that are uncorrelated with the outcome are presented in the supplemental material. In general, we find that including covariates that are uncorrelated with the outcome has a negligible impact on the treatment selection outcome and the final inference. On the contrary, including covariates that are moderately or highly correlated with the outcome can increase the chance of selecting the truly effective intervention to continue to the second stage of AMADs, increase the study power, and reduce the bias and MSE of estimators of the treatment effect. Moreover, the FWER is controlled at the nominal level when covariates are adjusted via the regression approach. The UMVCUE also has good properties in terms of being unbiased with only a relatively small increase in the MSE compared to the standard overall estimator of the treatment effect.

Our simulation study and the discussion in Section 5 also emphasise the importance of specifying the covariates in advance of the analyses. More specifically, adjusting for different sets of covariates in a single trial replication may lead to different treatment selection outcomes and different results of hypothesis testing. For treatment effect measures that have the inherit property of non-collapsibility, comparing adjusted results of different sets of covariates requires care. This is because extra analysis steps to marginalise the treatment effect estimates are necessary to ensure that the estimates are comparable. To avoid data dredging, having a statistical analysis plan in place prior to any of the data analyses is crucial. Such a practice also maintains the integrity of randomised studies.

Like any other randomized control trials, the sample size calculation of AMADs assuming the absence of covariates at the design stage is an approximation. The gain in power is maximised with covariate adjustment when all other assumptions made in the sample size calculation hold, e.g., dropout rate, the values of the nuisance parameter and the treatment effect under the (global) alternative hypothesis. Alternatively, one may perform simulation studies to identify an appropriate sample size given the information about the distribution of the covariates. This is still an approximation as there are uncertainties in the distributional assumptions.

We note that the benefits from implementing AMADs is affected by the recruitment rate and the length to observe the data that is used for treatment selection. In some health problems where the primary outcome is not available immediately, a surrogate (or a short-term) endpoint that is correlated with the long-term endpoint might be used for treatment selection. This has been one of the active research areas about AMADs.^17,57–60^ In this case, the probability of selecting the truly effective interventions can vary according to the correlation between the surrogate endpoint and the primary endpoint. When an uncorrelated (or weakly correlated) surrogate endpoint is used, there is a higher chance of selecting the truly ineffective interventions compared to the ideal case where the main outcome on all recruited patients is available for the interim analysis. We have not explored this but hypothesise that the role of covariates adjustment is similar in such a context.

We also have not incorporated randomization procedures in our simulation set-up but have assumed the ideal scenario where patients in each arm are comparable with respect to the baseline characteristics and other unmeasured factors. In practice, covariate imbalance can happen in a randomised trial due to the random nature in treatment allocation.^61^ A common practice is to adjust for the variables used in the randomisation procedures, e.g., minimisation, in the final analysis. This can improve the statistical power when the variables are predictive of the outcome.^62^ We believe chance imbalance has little impact on our general findings especially when a covariate-adaptive randomization procedure ^63^ is in place, the stage one sample size of AMADs is not small, and that the analyses adjust for the covariates used in the randomization procedure.

Another limitation of our investigation is that we have considered treatment selection without other adaptations, such as sample size re-estimation and adaptive randomization. We note that when an intervention is not selected to continue to stage two, there is no formal claim about its inference until a formal testing is conducted at the end of the study. The AMAD framework that is based on the group-sequential design accounts for this adaptation in a more direct sense and can include covariate adjusted for some of the commonly used models as shown by Jennison and Turnbull ^41^. We have also assumed that there is no missing data in our simulation study. Future work can explore how the missing data approaches may impact on AMADs when some of the covariates and responses are missing at random or missing not at random. Another direction could be exploring the sensitivity of some estimators in the presence of a population drift, especially when there are more than two stages. Note that we also have not provided the confidence interval for the UMVCUE in the case illustration in Section 4.3. This is because there are no established methods to construct valid confidence intervals based on the UMVCUE. More investigation is required to explore the construction of confidence intervals in the presence of covariates.

## Supplemental Material

sj-r-1-smm-10.1177_09622802221114544 - Supplemental material for The benefits of covariate adjustment for adaptive multi-arm designs Supplemental material, sj-R-1-smm-10.1177_09622802221114544 for The benefits of covariate adjustment for adaptive multi-arm designs by Kim May Lee, David S. Robertson, Thomas Jaki and Richard Emsley in Statistical Methods in Medical Research

sj-r-2-smm-10.1177_09622802221114544 - Supplemental material for The benefits of covariate adjustment for adaptive multi-arm designs Supplemental material, sj-R-2-smm-10.1177_09622802221114544 for The benefits of covariate adjustment for adaptive multi-arm designs by Kim May Lee, David S. Robertson, Thomas Jaki and Richard Emsley in Statistical Methods in Medical Research

## References

[1] Mahajan R Gupta K . Adaptive design clinical trials: Methodology, challenges and prospect. Indian J Pharmacol 2010; 42: 201. 20927243 10.4103/0253-7613.68417 PMC2941608

[2] Angus DC Alexander BM Berry S , et al. Adaptive platform trials: definition, design, conduct and reporting considerations. Nat Rev Drug Discov 2019; 18: 797–807. 10.1038/s41573-019-0034-3 31462747

[3] Saville BR Berry SM . Efficiencies of platform clinical trials: A vision of the future. Clinical Trials: Journal of the Society for Clinical Trials 2016; 13: 358–366. 26908536 10.1177/1740774515626362

[4] Pallmann P Bedding AW Choodari-Oskooei B et al. Adaptive designs in clinical trials: why use them, and how to run and report them. BMC Med 2018; 16: 1–15. 10.1186/s12916-018-1017-7 PMC5830330 29490655

[5] Hu F Rosenberger WF . The theory of response-adaptive randomization in clinical trials. Hoboken, New Jersey, USA: John Wiley & Sons, 2006.

[6] Rosenberger WF Sverdlov O Hu F . Adaptive randomization for clinical trials. J Biopharm Stat 2012; 22: 719–736. 22651111 10.1080/10543406.2012.676535

[7] Robertson DS Lee KM Lopez-Kolkovska BC , et al. Response-adaptive randomization in clinical trials: from myths to practical considerations. arXiv. 2020. Available from: http://arxiv.org/abs/2005.00564. 10.1214/22-STS865 PMC7614644 37324576

[8] Stallard N Todd S . Seamless phase II/III designs. Stat Methods Med Res 2011; 20: 623–634. 20724313 10.1177/0962280210379035

[9] Jaki T . Multi-arm clinical trials with treatment selection: what can be gained and at what price?. Clin Investig (Lond) 2015; 5: 393–399.

[10] Stallard N Todd S . Sequential designs for phase III clinical trials incorporating treatment selection. Stat Med 2003; 22: 689–703. 12587100 10.1002/sim.1362

[11] Kelly PJ Stallard N Todd S . An adaptive group sequential design for phase II/III clinical trials that select a single treatment from several. J Biopharm Stat 2005; 15: 641–658. 16022169 10.1081/BIP-200062857

[12] Stallard N Friede T . A group-sequential design for clinical trials with treatment selection. Stat Med 2008; 27: 6209–6227. 18792085 10.1002/sim.3436

[13] Urach S Posch M . Multi-arm group sequential designs with a simultaneous stopping rule. Stat Med 2016; 35: 5536–5550. 27550822 10.1002/sim.7077 PMC5157767

[14] Bauer P Kohne K . Evaluation of experiments with adaptive interim analyses. Biometrics 1994; 50 1029–1041. 7786985

[15] Bauer P Kieser M . Combining different ph+ases in the development of medical treatments within a single trial. Stat Med 1999; 18: 1833–1848. 10407255 10.1002/(sici)1097-0258(19990730)18:14<1833::aid-sim221>3.0.co;2-3

[16] Bretz F Schmidli H König F , et al. Confirmatory seamless phase II/III clinical trials with hypotheses selection at interim: general concepts. Biometrical Journal: Journal of Mathematical Methods in Biosciences 2006; 48: 623–634. 10.1002/bimj.200510232 16972714

[17] Friede T Parsons N Stallard N et al. Designing a seamless phase II/III clinical trial using early outcomes for treatment selection: an application in multiple sclerosis. Stat Med 2011; 30: 1528–1540. 21341301 10.1002/sim.4202

[18] Koenig F Brannath W Bretz F , et al. Adaptive Dunnett tests for treatment selection. Stat Med 2008; 27: 1612–1625. 17876763 10.1002/sim.3048

[19] Sargent DJ Goldberg RM . A flexible design for multiple armed screening trials. Stat Med 2001; 20: 1051–1060. 11276035 10.1002/sim.704

[20] Royston P Parmar MK Qian W . Novel designs for multi-arm clinical trials with survival outcomes with an application in ovarian cancer. Stat Med 2003; 22: 2239–2256. 12854091 10.1002/sim.1430

[21] Sampson AR Sill MW . Drop-the-losers design: normal case. Biometrical Journal: Journal of Mathematical Methods in Biosciences 2005; 47: 257–268. 10.1002/bimj.200410119 16053251

[22] Wason J Stallard N Bowden J , et al. A multi-stage drop-the-losers design for multi-arm clinical trials. Stat Methods Med Res 2017 feb; 26: 508–524. 25228636 10.1177/0962280214550759 PMC5302074

[23] Friede T Stallard N . A comparison of methods for adaptive treatment selection. Biometrical Journal: Journal of Mathematical Methods in Biosciences 2008; 50: 767–781. 10.1002/bimj.200710453 18932136

[24] Proschan MA Dodd LE . A modest proposal for dropping poor arms in clinical trials. Stat Med 2014; 33: 3241–3252. 24757049 10.1002/sim.6169 PMC4663989

[25] Bowden J Glimm E . Unbiased estimation of selected treatment means in two-stage trials. Biometrical J: J Math Methods Biosci 2008; 50: 515–527. 10.1002/bimj.200810442 18663760

[26] Carreras M Brannath W . Shrinkage estimation in two-stage adaptive designs with midtrial treatment selection. Stat Med 2013; 32: 1677–1690. 22744936 10.1002/sim.5463

[27] Kimani PK Todd S Stallard N . Conditionally unbiased estimation in phase II/III clinical trials with early stopping for futility. Stat Med 2013; 32: 2893–2910. 23413228 10.1002/sim.5757 PMC3813981

[28] Robertson DS Prevost AT Bowden J . Unbiased estimation in seamless phase II/III trials with unequal treatment effect variances and hypothesis-driven selection rules. Stat Med 2016; 35: 3907–3922. 27103068 10.1002/sim.6974 PMC5026174

[29] Stallard N Kimani PK . Uniformly minimum variance conditionally unbiased estimation in multi-arm multi-stage clinical trials. Biometrika 2018; 105: 495–501.

[30] Whitehead J Desai Y Jaki T . Estimation of treatment effects following a sequential trial of multiple treatments. Stat Med 2020; 39: 1593–1609. 32207166 10.1002/sim.8497 PMC7217198

[31] Robertson DS Choodari-Oskooei B Dimairo M , et al. Point estimation for adaptive trial designs; 2021. Available from: http://arxiv.org/abs/2105.08836. 10.1002/sim.9605 PMC7613995 36451173

[32] Posch M Koenig F Branson M , et al. Testing and estimation in flexible group sequential designs with adaptive treatment selection. Stat Med 2005; 24: 3697–3714. 16320264 10.1002/sim.2389

[33] Robinson LD Jewell NP . Some surprising results about covariate adjustment in logistic regression models. International Statistical Review/Revue Internationale de Statistique 1991; 59: 227–240.

[34] Kahan BC Jairath V Doré CJ , et al. The risks and rewards of covariate adjustment in randomized trials: an assessment of 12 outcomes from 8 studies. Trials 2014; 15: 1–7. 24755011 10.1186/1745-6215-15-139 PMC4022337

[35] Colantuoni E Rosenblum M . Leveraging prognostic baseline variables to gain precision in randomized trials. Stat Med 2015; 34: 2602–2617. 25872751 10.1002/sim.6507 PMC5018399

[36] Wang B Ogburn EL Rosenblum M . Analysis of covariance in randomized trials: More precision and valid confidence intervals, without model assumptions. Biometrics 2019; 75: 1391–1400. 31009064 10.1111/biom.13062

[37] Benkeser D Díaz I Luedtke A , et al. Improving precision and power in randomized trials for COVID-19 treatments using covariate adjustment, for binary, ordinal, and time-to-event outcomes. Biometrics 2021; 77:1467–1481. 10.1111/biom.13377 PMC7537316 32978962

[38] Jaki T Magirr D . Considerations on covariates and endpoints in multi-arm multi-stage clinical trials selecting all promising treatments. Stat Med 2013; 32: 1150–1163. 23112135 10.1002/sim.5669

[39] Friede T Kieser M . Blinded sample size recalculation for clinical trials with normal data and baseline adjusted analysis. Pharm Stat 2011; 10: 8–13. 19943322 10.1002/pst.398

[40] Zimmermann G Kieser M Bathke AC . Sample size calculation and blinded recalculation for analysis of covariance models with multiple random covariates. J Biopharm Stat 2020; 30: 143–159. 31327284 10.1080/10543406.2019.1632871

[41] Jennison C Turnbull BW . Group-sequential analysis incorporating covariate information. J Am Stat Assoc 1997; 92: 1330–1341.

[42] Zhao W Ma W Wang F , et al. Incorporating covariates information in adaptive clinical trials for precision medicine. Pharm Stat 2022; 21:176–195. 10.1002/pst.2160 34369053

[43] Donohue JF Fogarty C Lotvall J et al. Once-daily bronchodilators for chronic obstructive pulmonary disease: indacaterol versus tiotropium. Am J Respir Crit Care Med 2010; 182: 155–162. 20463178 10.1164/rccm.200910-1500OC

[44] Parsons N Friede T Todd S et al. An R package for implementing simulations for seamless phase II/III clinical trials using early outcomes for treatment selection. Comput Stat Data Anal 2012; 56: 1150–1160.

[45] The European Medicines Evaluation Agency . ICH topic E9: Statistical principles for clinical trials. 1998 https://www.ema.europa.eu/en/documents/scientific-guideline/ich-e-9-statistical-principles-clinical-trials-step-5_en.pdf

[46] The European Medicines Evaluation Agency . Guideline on multiplicity issues in clinical trials (draft). 2017. https://www.ema.europa.eu/en/documents/scientific-guideline/draft-guideline-multiplicity-issues-clinical-trials_en.pdf

[47] US Food and Drug Administration. Guidance for industry on multiple endpoints in clinical trials. 2017. https://wwwfdagov/downloads/drugs/guidancecomplianceregulatoryinformation/guidances/ucm536750pdf.

[48] Dunnett CW . A multiple comparison procedure for comparing several treatments with a control. J Am Stat Assoc 1955; 50: 1096–1121.

[49] Grayling MJ Wason JM . A web application for the design of multi-arm clinical trials. BMC Cancer 2020; 20: 1–12. 10.1186/s12885-020-6525-0 PMC6995188 32005187

[50] Sill MW Sampson AR . Extension of a two-stage conditionally unbiased estimator of the selected population to the bivariate normal case. Communications in Statistics–Theory and Methods 2007; 36: 801–813.

[51] Marschner IC . A general framework for the analysis of adaptive experiments. Stat Sci 2021; 36: 465–492.

[52] Magirr D Jaki T Posch M , et al. Simultaneous confidence intervals that are compatible with closed testing in adaptive designs. Biometrika 2013; 100: 985–996. 27019516 10.1093/biomet/ast035 PMC4806862

[53] Robertson DS Prevost AT Bowden J . Accounting for selection and correlation in the analysis of two-stage genome-wide association studies. Biostatistics 2016; 17: 634–649. 26993061 10.1093/biostatistics/kxw012 PMC5031943

[54] R Core Team. R: A Language and Environment for Statistical Computing. Vienna, Austria; 2021. Available from: https://www.R-project.org/.

[55] Daniel R Zhang J Farewell D . Making apples from oranges: Comparing noncollapsible effect estimators and their standard errors after adjustment for different covariate sets. Biom J 2021; 63: 528–557. 33314251 10.1002/bimj.201900297 PMC7986756

[56] Groenwold RH Moons KG Peelen LM , et al. Reporting of treatment effects from randomized trials: A plea for multivariable risk ratios. Contemp Clin Trials 2011; 32: 399–402. 21195797 10.1016/j.cct.2010.12.011

[57] Stallard N . A confirmatory seamless phase II/III clinical trial design incorporating short-term endpoint information. Stat Med 2010; 29: 959–971. 20191605 10.1002/sim.3863

[58] Kunz CU Friede T Parsons N , et al. A comparison of methods for treatment selection in seamless phase II/III clinical trials incorporating information on short-term endpoints. J Biopharm Stat 2015; 25: 170–189. 24697322 10.1080/10543406.2013.840646 PMC4339952

[59] Stallard N Kunz CU Todd S , et al. Flexible selection of a single treatment incorporating short-term endpoint information in a phase II/III clinical trial. Stat Med 2015; 34: 3104–3115. 26112909 10.1002/sim.6567 PMC4745001

[60] Jörgens S Wassmer G König F , et al. Nested combination tests with a time-to-event endpoint using a short-term endpoint for design adaptations. Pharm Stat 2019; 18: 329–350. 30652401 10.1002/pst.1926

[61] Senn SJ . Covariate imbalance and random allocation in clinical trials. Stat Med 1989; 8: 467–475. 2727470 10.1002/sim.4780080410

[62] Thompson DD Lingsma HF Whiteley WN , et al. Covariate adjustment had similar benefits in small and large randomized controlled trials. J Clin Epidemiol 2015; 68: 1068–1075. 25497979 10.1016/j.jclinepi.2014.11.001 PMC5708297

[63] Lin Y Zhu M Su Z . The pursuit of balance: an overview of covariate-adaptive randomization techniques in clinical trials. Contemp Clin Trials 2015; 45: 21–25. 26244705 10.1016/j.cct.2015.07.011

