# Supplementary material for: The benefits of covariate adjustment for adaptive multi-arm designs
Source: Stat Methods Med Res. Author manuscript; Available in PMC 2022 Nov 12. (PMC7613816; doi:10.1177/09622802221114544)
Supplement: Supplementary file 1 [file EMS153901-supplement-Supplementary_File.pdf]

Supplemental material

Additional simulation output of Section 4

Table S1: The likelihood of selecting a treatment at stage one when one of the four analysis approaches is used. The numbers show the minimum (Min.), maximum (Max.) and the difference between the maximum and min probability (Diff.)

	$\rho_1 = 0$ $\rho_2 = 0$	$\rho_1 = 0$ $\rho_2 = 0.2$	$\rho_1 = 0.2$ $\rho_2 = 0.2$	$\rho_1 = 0.1$ $\rho_2 = 0.6$	$\rho_1 = 0$ $\rho_2 = 0$	$\rho_1 = 0$ $\rho_2 = 0.2$	$\rho_1 = 0.2$ $\rho_2 = 0.2$	$\rho_1 = 0.1$ $\rho_2 = 0.6$
	P(select T1)				P(select T2)			
	LFC 50							
Min.	0.0606	0.0568	0.0537	0.0254	0.9393	0.9392	0.9400	0.9397
Max.	0.0607	0.0608	0.0600	0.0603	0.9394	0.9432	0.9463	0.9746
Diff.	0.0001	0.0039	0.0064	0.0349	0.0001	0.0039	0.0063	0.0349
	LFC 80							
Min.	0.0160	0.0144	0.0130	0.0035	0.9837	0.9841	0.9841	0.9840
Max.	0.0163	0.0159	0.0159	0.0160	0.9840	0.9856	0.9870	0.9965
Diff.	0.0003	0.0014	0.0029	0.0125	0.0003	0.0014	0.0029	0.0125
	Step 50							
Min.	0.2158	0.2112	0.2073	0.1622	0.7833	0.7842	0.7848	0.7834
Max.	0.2167	0.2158	0.2152	0.2166	0.7842	0.7888	0.7927	0.8378
Diff.	0.0009	0.0047	0.0079	0.0544	0.0009	0.0047	0.0079	0.0544
	Step 80							
Min.	0.1387	0.1341	0.1303	0.0875	0.8600	0.8599	0.8602	0.8592
Max.	0.1399	0.1401	0.1398	0.1408	0.8613	0.8659	0.8697	0.9125
Diff.	0.0012	0.0060	0.0095	0.0533	0.0012	0.0060	0.0095	0.0533

Simulation output with additional six covariates

This section presents the trial operating characteristics of AMAD when the data generating mechanisms have an additional six covariates that are uncorrelated with the outcome. More specifically, there are a total of eight covariates where $X1_k$ and $X2_k$ have correlation values as considered in Section 4 but the other six covariates have zero correlation. Other simulation settings are the same as in Section 4. An

additional comparator is the regression model that adjusts for all the eight covariates in the analysis, denoted by $Adjusted_{all}$ in the following results.

Figure S1 shows the treatment selection frequency when comparing an adjusted approach with the unadjusted approach. Figure S2 shows the unconditional rejection probabilities from the two-stage AMAD. We find that the new comparator, $Adjusted_{all}$ has similar performance to the $Adjusted_{X2}$. Table S2 shows that FWER is controlled (strongly) by all the analysis approaches. Table S3 shows the likelihood of selecting a treatment at stage one when one of the five analysis approaches is used.

Table S2: Probability of hypothesis rejections under the null scenario for each DGM when each analysis approach is implemented. The Monte-Carlo simulation error for the nominal FWER is $\sqrt{0.025 * 0.975/100000}$ and the 95% coverage for the nominal FWER is $[0.02403, 0.02597]$.

	$\rho_1 = 0$, $\rho_2 = 0$	$\rho_1 = 0$, $\rho_2 = 0.2$	$\rho_1 = 0.2$, $\rho_2 = 0.2$	$\rho_1 = 0.1$, $\rho_2 = 0.6$
Approach	FWER			
Unadjusted	0.02546	0.02548	0.02553	0.02493
Adjusted$_{X1}$	0.02541	0.02562	0.02554	0.02539
Adjusted$_{X2}$	0.02557	0.02530	0.02498	0.02568
Adjusted$_{both}$	0.02537	0.02514	0.02516	0.02550
Adjusted$_{all}$	0.02542	0.02535	0.02560	0.02543
	Probability of rejecting H_{01}			
Unadjusted	0.01284	0.01279	0.01285	0.01256
Adjusted$_{X1}$	0.01278	0.01297	0.01285	0.01270
Adjusted$_{X2}$	0.01290	0.01284	0.01257	0.01304
Adjusted$_{both}$	0.01284	0.01277	0.01270	0.01322
Adjusted$_{all}$	0.01297	0.01292	0.01308	0.01312
	Probability of rejecting H_{02}			
Unadjusted	0.01262	0.01269	0.01268	0.01237
Adjusted$_{X1}$	0.01263	0.01265	0.01269	0.01269
Adjusted$_{X2}$	0.01267	0.01246	0.01241	0.01264
Adjusted$_{both}$	0.01253	0.01237	0.01246	0.01228
Adjusted$_{all}$	0.01245	0.01243	0.01252	0.01231

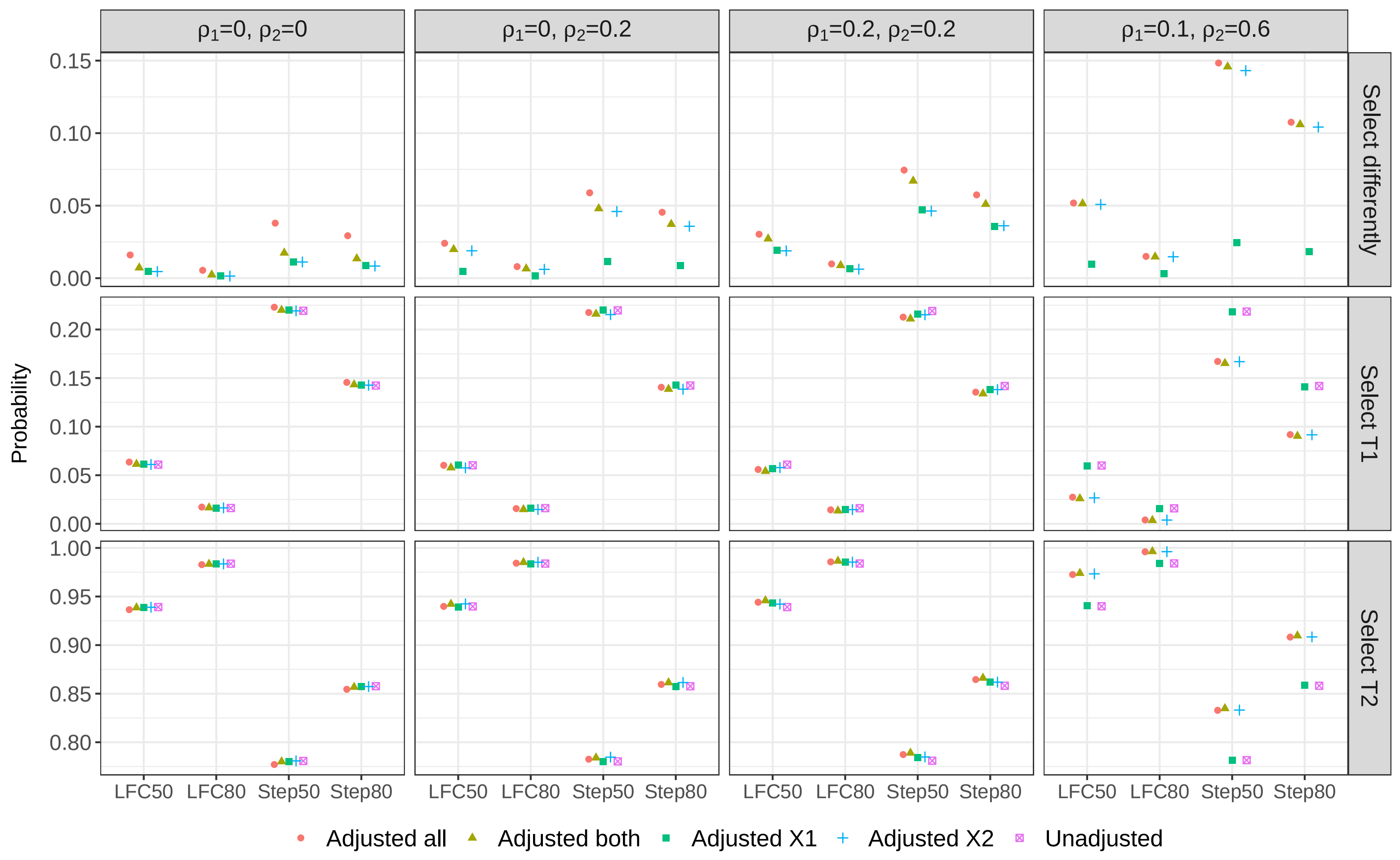

Figure S1: Treatment selection frequency/ probability: when an adjusted approach has a different selection result to the unadjusted approach (first panel), when both an adjusted approach and the unadjusted approach select T1 (second panel), and when both an adjusted approach and the unadjusted approach select T2 (third panel).

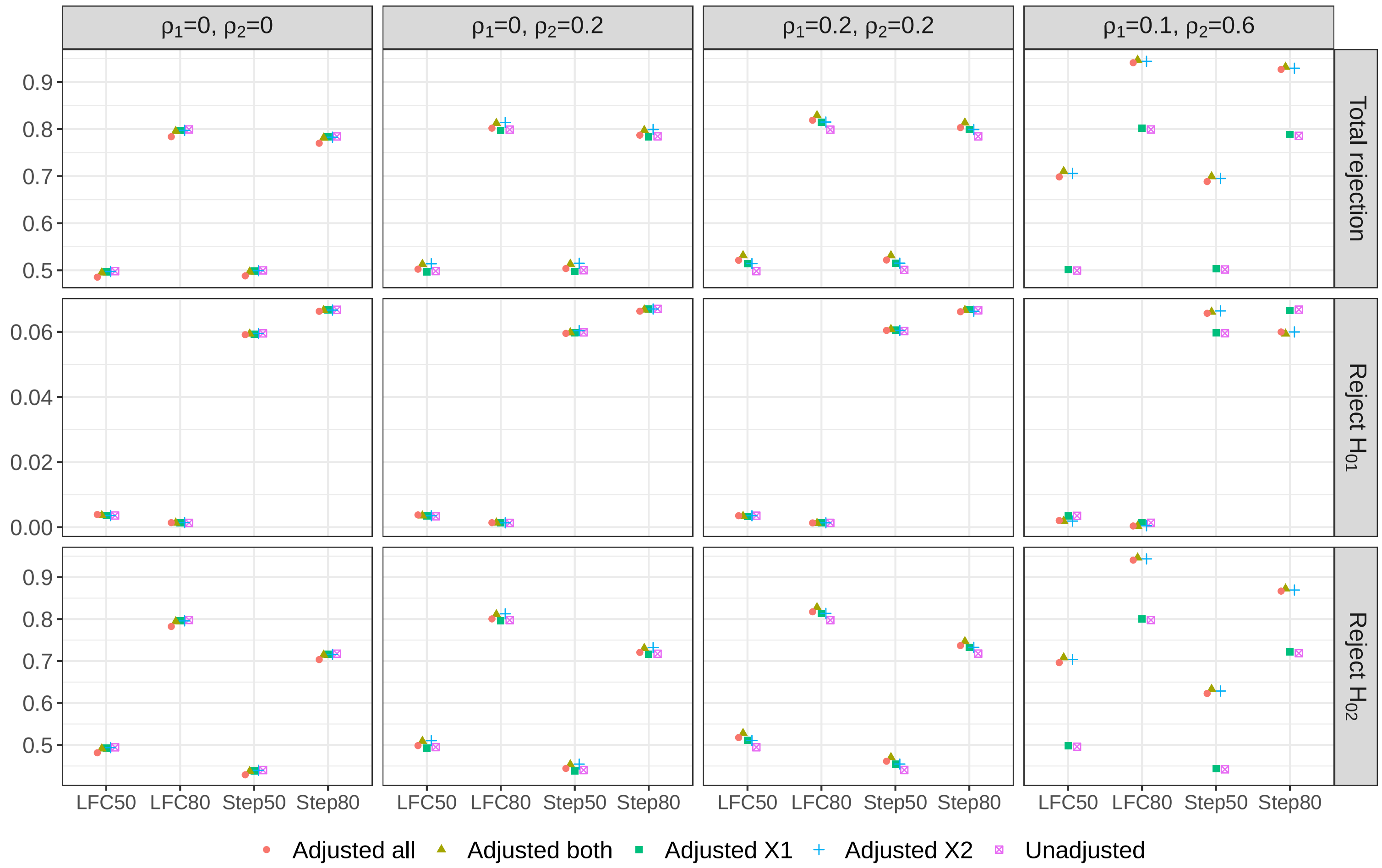

Figure S2: Unconditional rejection probabilities from the two-stage AMAD: when anyone hypothesis is rejected (first panel), when H_{01} is rejected (second panel), and when H_{02} is rejected (third panel).

Table S3: The likelihood of selecting a treatment at stage one when one of the five analysis approaches is used. The numbers show the minimum (Min.), maximum (Max.) and the difference between the maximum and min probability (Diff.)

	$\rho_1 = 0$ $\rho_2 = 0$	$\rho_1 = 0$ $\rho_2 = 0.2$	$\rho_1 = 0.2$ $\rho_2 = 0.2$	$\rho_1 = 0.1$ $\rho_2 = 0.6$	$\rho_1 = 0$ $\rho_2 = 0$	$\rho_1 = 0$ $\rho_2 = 0.2$	$\rho_1 = 0.2$ $\rho_2 = 0.2$	$\rho_1 = 0.1$ $\rho_2 = 0.6$
	P(select T1)				P(select T2)			
	LFC 50							
Min.	0.0609	0.0575	0.0542	0.0260	0.9365	0.9394	0.9391	0.9400
Max.	0.0635	0.0606	0.0609	0.0600	0.9391	0.9424	0.9458	0.9740
Diff.	0.0027	0.0031	0.0067	0.0340	0.0027	0.0031	0.0067	0.0340
	LFC 80							
Min.	0.0162	0.0147	0.0134	0.0036	0.9828	0.9837	0.9840	0.9841
Max.	0.0172	0.0163	0.0160	0.0159	0.9838	0.9853	0.9866	0.9964
Diff.	0.0010	0.0015	0.0026	0.0123	0.0010	0.0015	0.0026	0.0123
	Step 50							
Min.	0.2192	0.2153	0.2110	0.1652	0.7771	0.7800	0.7810	0.7816
Max.	0.2229	0.2200	0.2190	0.2184	0.7808	0.7847	0.7890	0.8348
Diff.	0.0037	0.0047	0.0080	0.0532	0.0037	0.0047	0.0080	0.0532
	Step 80							
Min.	0.1423	0.1386	0.1339	0.0903	0.8545	0.8571	0.8582	0.8581
Max.	0.1455	0.1429	0.1418	0.1419	0.8577	0.8614	0.8661	0.9097
Diff.	0.0032	0.0044	0.0079	0.0516	0.0032	0.0043	0.0079	0.0515
